# A facile chemical synthesis of nanoflake NiS_2_ layers and their photocatalytic activity

**DOI:** 10.1039/d2ra01067d

**Published:** 2022-04-05

**Authors:** Mohammed M. Gomaa, Mohamed H. Sayed, Mahmoud S. Abdel-Wahed, Mostafa Boshta

**Affiliations:** Solid State Physics Department, National Research Centre 12622 Dokki Giza Egypt dr.metwalyg@gmail.com +20-1272110812; Molecular and Fluorescence Spectroscopy Lab., Central Laboratories Network, National Research Centre 12622 Dokki Giza Egypt; Water Pollution Research Department, National Research Centre 12622 Dokki Giza Egypt

## Abstract

A single-phase and crystalline NiS_2_ nanoflake layer was produced by a facile and novel approach consisting of a two-step growth process. First, a Ni(OH)_2_ layer was synthesized by a chemical bath deposition approach using a nickel precursor and ammonia as the starting solution. In a second step, the obtained Ni(OH)_2_ layer was transformed into a NiS_2_ layer by a sulfurization process at 450 °C for 1 h. The XRD analysis showed a single-phase NiS_2_ layer with no additional peaks related to any secondary phases. Raman and X-ray photoelectron spectroscopy further confirmed the formation of a single-phase NiS_2_ layer. SEM revealed that the NiS_2_ layer consisted of overlapping nanoflakes. The optical bandgap of the NiS_2_ layer was evaluated with the Kubelka–Munk function from the diffuse reflectance spectrum (DRS) and was estimated to be around 1.19 eV, making NiS_2_ suitable for the photodegradation of organic pollutants under solar light. The NiS_2_ nanoflake layer showed photocatalytic activity for the degradation of phenol under solar irradiation at natural pH 6. The NiS_2_ nanoflake layer exhibited good solar light photocatalytic activity in the photodegradation of phenol as a model organic pollutant.

## Abstract 2

A single phase nanoflake NiS_2_ layer synthesized by a facile chemical bath deposition showed good solar light photocatalytic degradation of phenol with good stability and reusability.

## Graphical abstract

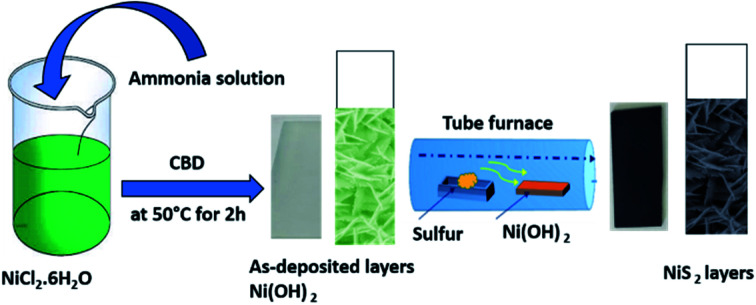

## Introduction

In the last decades, nanostructured transition metal sulfides (NTMSs) have received considerable attention in different fields because of their unique optical, magnetic and catalytic properties.^1–3^ The properties of these materials are strongly dependent on the dimension, size, and morphologies of fabricated materials,^4–6^ making these materials very promising for numerous advanced applications such as adsorbents for dye removal,^7^ supercapacitors,^8^ rechargeable lithium-ion batteries,^9^ hydrodesulfurization catalysts,^10^ hydrogen evolution reaction,^4,11,12^ and catalysts in the degradation of organic dyes.^13^

Metal sulfide materials such as zinc sulfide,^14^ manganese sulfide,^15^ silver sulfide,^16^ iron sulfide,^17^ molybdenum sulfide,^18^ nickel sulfide,^12^ and copper sulfides, have been reported and studied extensively.^19^ Among the metal sulfides, nickel sulfides are more favorable in terms of earth-abundant resources, forming numerous phases such as NiS, NiS_2_, Ni_3_S_2_, Ni_3_S_4_, Ni_7_S_6_, and Ni_9_S_8_, which are suitable as alternative materials for different applications.^20–23^ Nickel disulfide (NiS_2_) crystallizes in a pyrite-like structure (FeS_2_), with a cubic phase *Pa*3̄ symmetry.^10,24,25^ Nanostructured pyrite NiS_2_ with a cubic structure has interesting optical, electronic, and magnetic properties.^3,10,26^ NiS_2_ nanostructures with controlled morphology such as nanoparticles, nanowires, nanosheets and hollow microspheres,^27–29^ have been considered as promising semiconducting materials for catalytic applications due to their low-cost, nontoxicity and chemical stability.^30,31^ However, the catalytic performance of NiS_2_ in the degradation of organic pollutants such as endocrine disrupting compounds (EDCs) is still less competitive compared to other catalytic materials based on phosphides and noble metals.^4,13^ In this regard, numerous techniques have been used to develop and fabricate nickel sulfide nanostructures with good physical and chemical properties including hydrothermal methods,^23,32,33^ solvothermal,^34^ decomposition of single-source precursors,^35^ microwave-assisted synthesis,^36,37^ solventless route in air,^38^ sonochemical,^39^ and ultrasonic spray pyrolysis.^10^ Most of these methods are suitable for preparing nickel sulfides in powder form with other different phases sometimes accompanying.^22,29,38^ The different possible phases of nickel sulfide make the synthesis of single-phase nickel disulfide very complicated.^20,29,34^ Therefore, the demand for an alternative approach to prepare a single phase of nickel disulfide layers with a high specific area and uniform morphology is still a major challenge, and will open doors to various opportunities for advanced applications.

Endocrine disrupting compounds (EDCs) such as phenol and its derivatives are a category of dangerous persistent organic pollutants, which are usually present in low concentrations in water environments. Phenol molecules are considered very harmful to human health, marine creatures and living organisms due to their carcinogenic, mutagenic, stability, and bioaccumulation nature, even in low concentrations. Phenolic compounds are discharged to ecology through effluent from many industries for instance, paint production, processing of petroleum, tanning, and pharmaceuticals.^40–42^ The conventional treatment is not very effective for the removal of these hazardous pollutants. Thus, the development of novel techniques is essential to address this issue. Morphological control is one of the effective approaches for promoting the photodegradation of phenol using NiS_2_ with nanoflake morphology. One of the big problems in the photocatalyst process is separation and recovery restriction of the photocatalyst from effluent after the treatment process. Therefore, herein, this problem is overcome through immobilizing prepared nickel sulfide on a glass substrate as layers.

In this study, single-phase NiS_2_ nanoflake layers were successfully processed *via* a facile two-step fabrication process. First, Ni(OH)_2_ nanoflake layers were grown on glass substrates by the chemical bath deposition method, followed by the phase transformation of Ni(OH)_2_ into NiS_2_*via* a sulfurization process. Structural, morphological and optical properties as well as the catalytic activity of the obtained NiS_2_ layer were studied. The unique nanoflake-like morphology of NiS_2_ serves as an efficient photocatalyst for the degradation of destructive organic pollutants (phenol as the model organic compound).

## Experimental

### NiS_2_ layers deposition

**Fig. 1:**
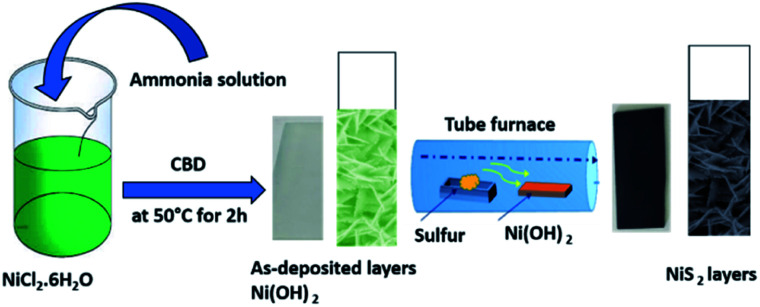
Schematic description of the synthesis of porous NiS_2_ nanoflake layers.

First, the chemical bath deposition (CBD) approach was used for the synthesis of the nanoflake-structured nickel hydroxide layer. The glass substrates were ultrasonically cleaned using acetone and ethyl alcohol for 20 min followed by distilled water, then dried with nitrogen gas, prior to loading into the reaction bath. 0.1 M aqueous solution of nickel chloride (NiCl_2_·6H_2_O – Sigma Aldrich) and ammonia solution were used as the source of Ni^2+^ and complexing agent for layer deposition, respectively. In a typical experimental procedure, the ammonia solution was added drop wise into the nickel chloride solution under continuous magnetic stirring to produce a clear and homogeneous aqueous solution as the starting solution. The precleaned glass substrates were vertically immersed in the solution bath at optimum deposition temperature, (*T*_d_ = 50 °C) and pH 11 during the synthesis process. After 2 h, the blue-colored solution changed to a greenish white color with the formation of Ni(OH)_2_ layer on the surface of the substrate by the adsorption and nucleation of the nickel cations on the substrate. The as-deposited Ni(OH)_2_ layers were transferred into a tube furnace with excessive sulfur powder and subsequently sulfurized at 450 °C for 1 h in nitrogen atmosphere to obtain a nickel sulfide layer.^43,44^ For the ease of understanding, the facile CBD and synthesis process for the nanoflake structured NiS_2_ layer is schematically described in Fig. 1.

The formation mechanism of the nanoflake-structured nickel disulfide layer is divided into two processes: first, the formation of a nickel hydroxide phase *via* the chemical bath deposition route, as indicated by the following equations:

1 Ni^2+^ + 2OH^−^ → Ni(OH)_2_↓

2 Ni(OH)_2_ + 4NH_3_ → [Ni(NH_3_)]^4+^ + 2OH^−^

3 [Ni(NH_3_)]^4+^ + 2OH^−^ → Ni(OH)_2_↓ + 4NH_3_↑

The detailed mechanism of the formation of nickel hydroxide by chemical bath deposition (CBD) can be found elsewhere.^45,46^

Second, the transformation of as deposited Ni(OH)_2_ layers to nickel disulfide^2^ due to reaction of Ni(OH)_2_ with sulfur atoms at 450 °C according to the following reactions:

4 Ni(OH)_2_ + S ↔ NiS + 2OH^−^

5 NiS + S → NiS_2_

### Characterization of prepared immobilized NiS_2_

In this study, the structural investigation and phase identification of the as-prepared NiS_2_ layer was analyzed by X-ray powder diffraction (XRD) with a Panalytical X'Pert diffractometer using Cu Kα1 radiation at 45 kV and 40 mA. Scanning electron microscopy (SEM) (QUANTA FEG250) was used for the surface morphology imaging of the obtained layers. X-ray photoelectron spectroscopy (XPS) was collected on K-Alpha (Themo Fisher Scientific, USA) with monochromatic X-ray Al K-alpha radiation at pressure 10^−9^ mbar to determine the elemental composition and electronic states of the NiS_2_ layer. Raman analysis was performed on a confocal Raman microscope model WITec Alpha 300 RA under the laser excitation of 532 nm. Diffuse reflectance spectra were carried out using a UV/Vis/NIR spectrophotometer (Jasco V770) in the wavelength range 250–1000 nm.

### Evaluation of the photocatalytic performance of the as-prepared immobilized NiS_2_ layer

The photocatalytic performance of the as-prepared NiS_2_ layer was established by photodegradation of phenol as a model organic pollutant. For this purpose, the NiS_2_ slide is primarily fixed by a silicon adhesive on a 2 cm-height edge inside a 150 mL beaker. After that, 90 mL of 10 mg L^−1^ phenol solution was placed in dark and stirred by a magnetic stirrer for 30 min to achieve adsorption desorption equilibrium. The beaker was irradiated vertically in a solar system (UVA CUBE 400, Dr Hönle AG UV Technology, Germany) equipped with a halogen lamp (model: SOL 500), which is simulated to the natural sunlight (1000 W m^−2^). At definite time intervals, a 1 mL sample was withdrawn from the beaker and 1 mL double distilled water was inserted instead to keep the distance between the light source and meniscus of solution constant all over the experiment duration. Phenol concentration in the withdrawn samples was determined by a high-performance liquid chromatograph (HPLC, Agilent 1260, USA) equipped with an analytical column Zorbax reverse-phase C18 and a diode-array detector at 280 nm wavelength. Each point was measured in triplet and the average was recorded. The column temperature was kept at 25 °C during the analysis. Gradient elution was obtained using water (mobile phase A) and acetonitrile (mobile phase B). 75% A mobile phase was eluted for 1 min, and then decreased to 60% A for 2 min. The flow rate of the mobile phase was kept at 0.5 mL min^−1^. The generation of redox reactive species by NiS_2_ after solar irradiation excitation was inspected by 1 mmol ammonium oxalate (AO) as the hole (h^+^) scavenger agent, 1 mmol *para*-benzoquinon (*p*-BQ) as the superoxide radical (O_2_˙^−^) scavenger and 1 mmol isopropyl alcohol (IPA) as the hydroxyl radical (˙OH) scavenger.

## Results and discussion

### Structural and elemental composition properties

**Fig. 2:**
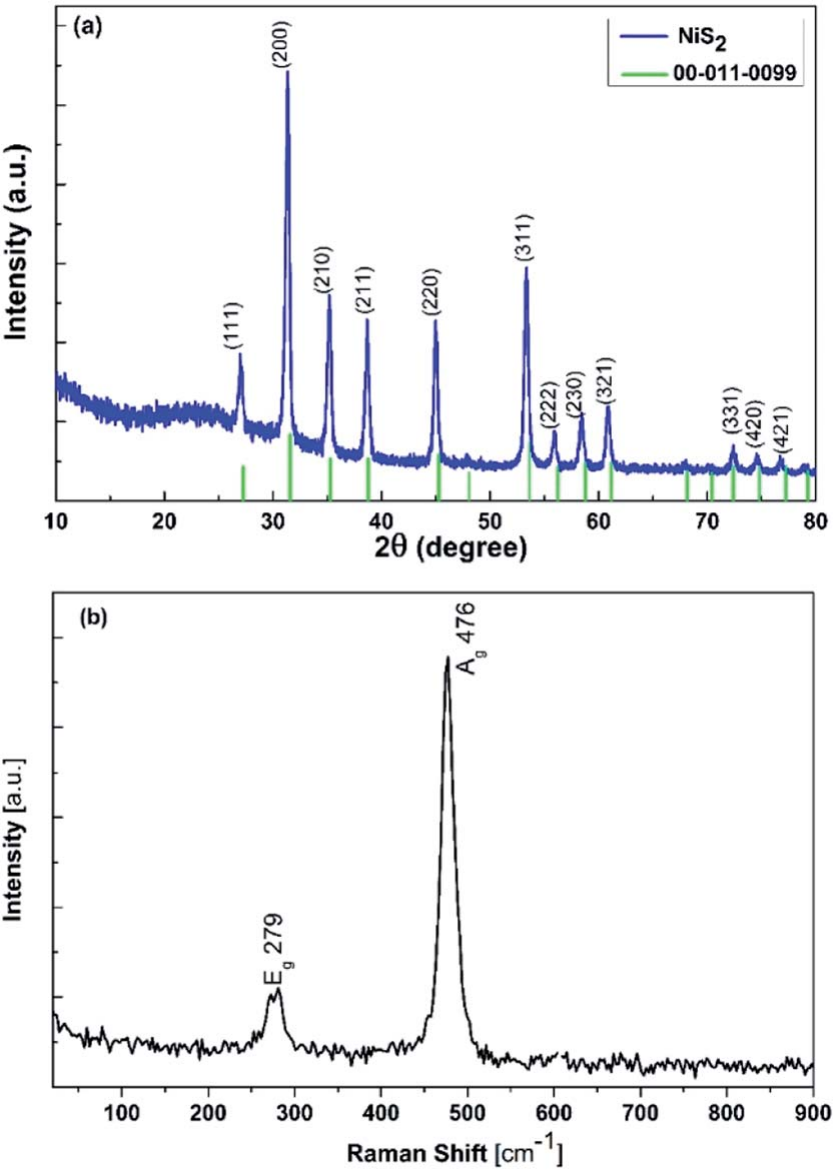
(a) XRD pattern and (b) Raman spectrum of nanoflake NiS_2_ layer.

The XRD and Raman data for the as-prepared NiS_2_ layer are presented in Fig. 2. The XRD pattern of the NiS_2_ layer (Fig. 2(a)) show sharp and dominant characteristic peaks of the NiS_2_ cubic structure (JCPDS card no. 00-011-0099),^45^ with no additional peaks related to any other crystalline nickel compounds such as nickel oxide, nickel hydroxide and other phases of nickel sulfides, indicating the complete transformation of the Ni(OH)_2_ phase to NiS_2_ phase.^47–49^ The XRD analysis well matched with reported studies in literature.^28,30,31,50^ The average crystallite size (*D*) of the NiS_2_ layer was calculated using the Scherrer–Debye formula (eqn (6)) for the (200) reflection plane.6
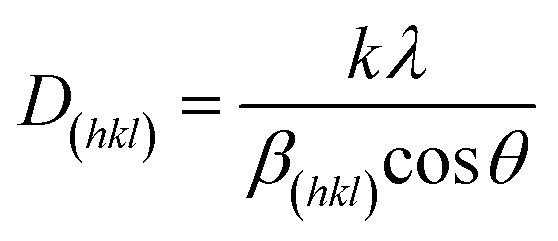
where *K* is the Debye constant, *λ* is the X-ray wavelength, *β* is the line broadening at full width at half maximum of the diffraction peak, and *θ* is the Bragg's angle.^51^ The calculated crystallite size of the NiS_2_ layers was approximately 26 nm.

The surface Raman spectrum measured at room temperature of the NiS_2_ layer (Fig. 2(b)) shows the dominant characteristic peaks of the NiS_2_ phase.^20^ The peaks at 279 and 476 cm^−1^ are assigned to *E*_g_ and *A*_g_ photons, respectively. The observed peaks shifted towards a lower frequency compared with the NiS_2_ single crystal. The obtained spectrum shows no noticeable characteristic peaks related to possible secondary phases and is consistent with previous reports.^11,28^

**Fig. 3:**
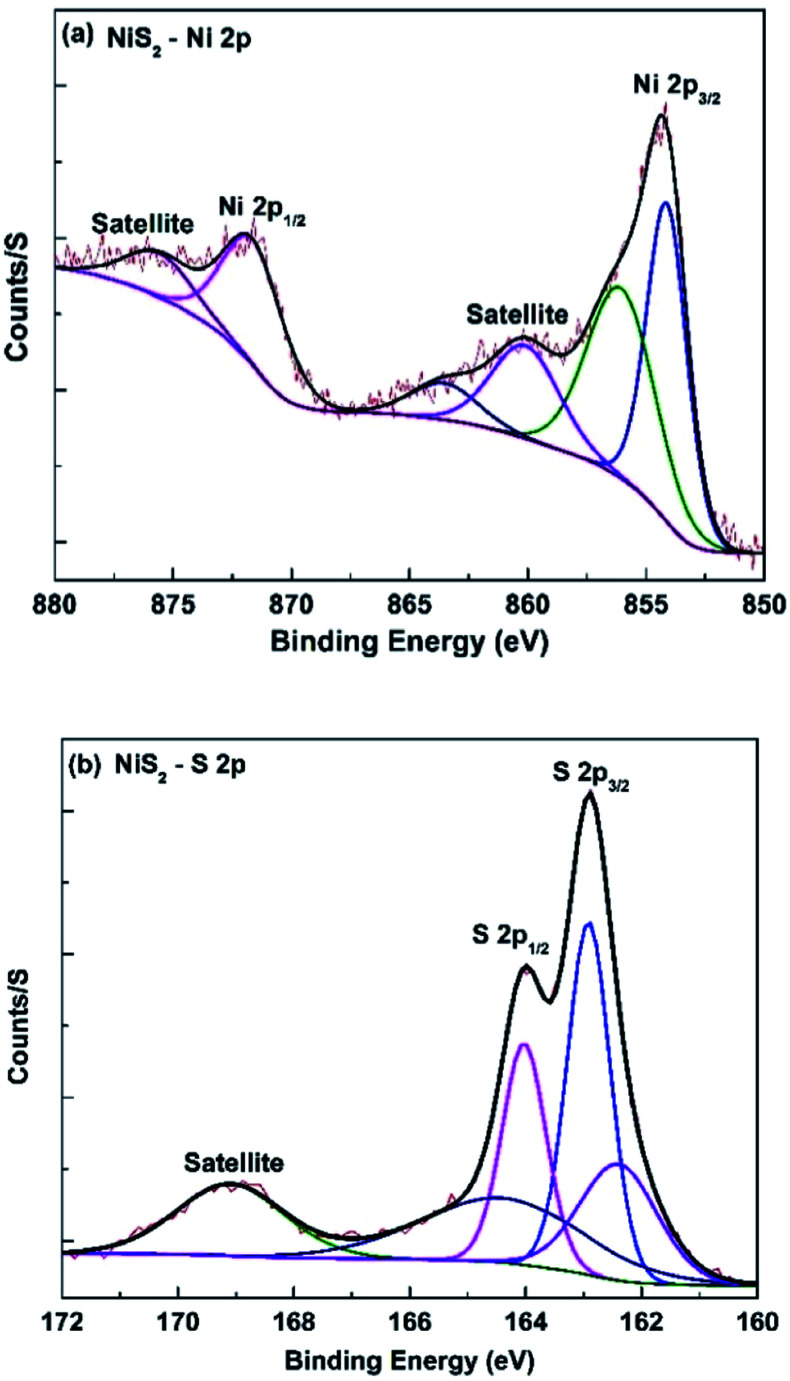
XPS spectra of nanoflake NiS_2_ layers: (a) Ni 2p and (b) S 2p.

In order to study the elemental compositions and electronic states of the nanoflake NiS_2_ layer, X-ray photoelectron spectroscopy (XPS) measurements were performed in this study. Fig. 3(a) presents the high-resolution XPS spectrum of Ni 2p for the nanostructured NiS_2_ layer, which has two main peaks appearing at 854.12 and 871.63 eV, fitting to the binding energy of Ni 2p_3/2_ and Ni 2p_1/2_, respectively. In addition, both Ni 2p_3/2_ and Ni 2p_1/2_ have shake-up satellite peaks located at 860.1 eV and 875.59 eV, respectively. Peak fitting analysis to separate overlapping peaks was made for the Ni 2p_3/2_ component, which indicates that it can be de-convoluted into a pair of peaks located at 854.12 and 856.05 eV, corresponding to Ni^2+^ and Ni^3+^ in NiS_2_, respectively. The existence of Ni^3+^ results from the surface oxidation of NiS_2_, which is in agreement with literature. The collected XPS results of the deconvolution of Ni 2p are in agreement with the reported binding energy values for Ni^2+^ and Ni^3+^.^4,36^ In addition, the spectral deconvolution of the S 2p spectrum (Fig. 3(b)) consists of two strong peaks at 162.91 (S 2p_3/2_) and 164.38 eV (S 2p_1/2_), implying the presence of unsaturated S atoms on the Ni–S and S–S bonds in NiS_2_. These results fit well with NiS_2_ single crystal XPS data.^22,29,52^

### Morphological properties

**Fig. 4:**
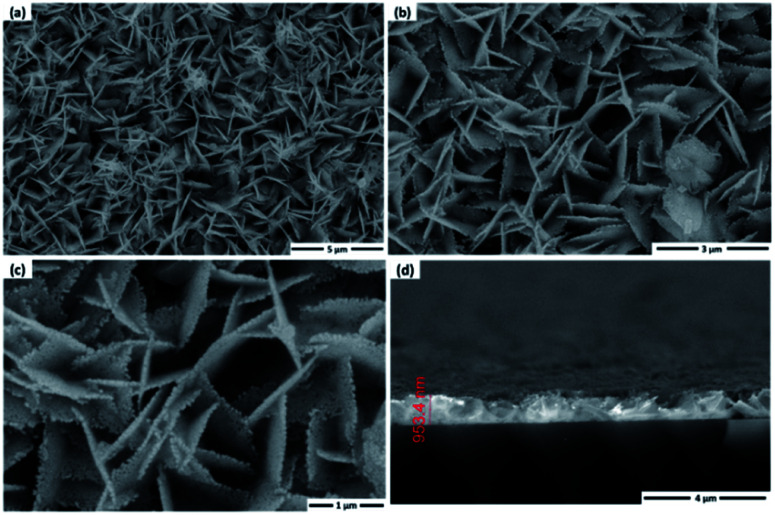
(a–c) SEM images of NiS_2_ with different magnifications, and (d) cross section of the NiS_2_ layer.

The morphology of the NiS_2_ layer was investigated by SEM. Fig. 4(a–d) shows the SEM images of the surface with different magnification, and cross section of the NiS_2_ layer, synthesized on the glass substrate. The top view images of the as-prepared NiS_2_ layer show that the surface of the NiS_2_ sample reveals a rough nanoflake-like structure, with homogeneous and uniform distribution as well as a pinhole free layer. Moreover, the magnified view images show that the cross-linked nanoflakes are compact and uniform, resulting in a network architecture on the substrates. Also, the rough nanoflake edges observed clearly in Fig. 4(c) can be associated with the sulfurization process of the as-deposited Ni(OH)_2_ layer as a result of gas release and dehydration during annealing, leading to the formation of NiS_2_ with a high surface area structure.^44^ The high surface area and rough morphology can significantly influence the photocatalytic performance of materials.^20,28^ A cross-sectional image (Fig. 4(d)) exhibits that the NiS_2_ layer has a uniform thickness in the range of approximately 950 nm.

### Optical properties

The energy bandgap of NiS_2_ was derived from the diffuse reflectance of the obtained layer using the Kubelka–Munk (KM) function^53,54^, as indicated by the following equation:7
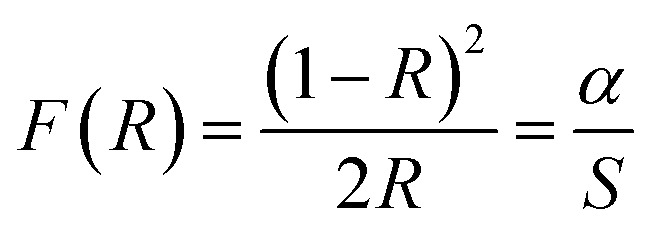
where *F*(*R*) is the (KM) function, *R* is the diffused reflectance, *α* is the absorption coefficient, and *S* is the scattering coefficient. The optical band gap energy (*E*_g_) of the NiS_2_ layer can be calculated by the Tauc's equation:^55^

8 *αhν* = *A*(*hν* − *E*_g_)^*n*^

where (*hν*) is the incident photon energy, *α* is the absorption coefficient, *A* is a constant, (*E*_g_) is the optical band gap energy. Based on the KM function and Tauc's equation, the optical bandgap energy of the NiS_2_ layer can be estimated using the following equation:

9 *F*(*R*)*hν* = *A*(*hν* − *E*_g_)^*n*^

**Fig. 5:**
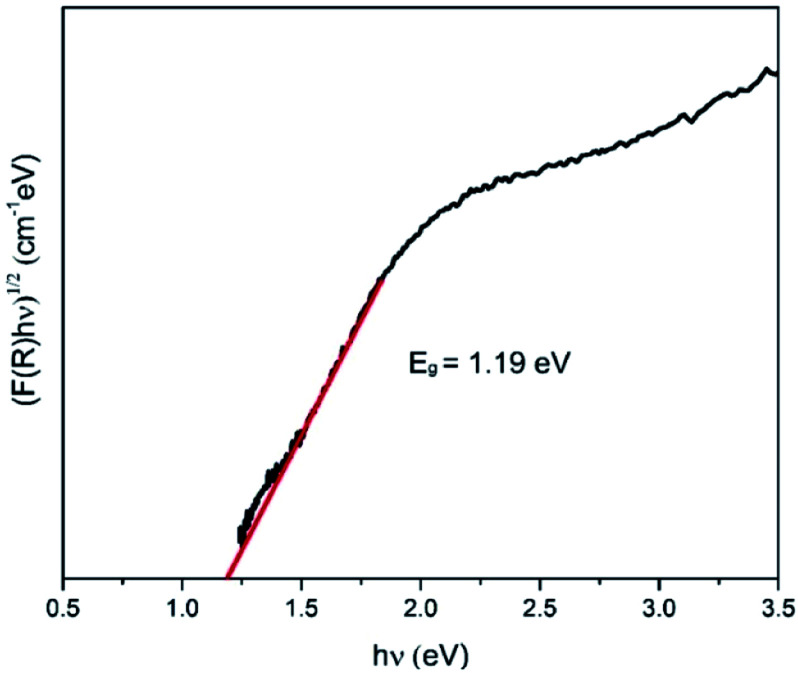
(*F*(*R*)*hν*)^1/2^*vs. hν* plot of the nanoflake NiS_2_ layer.

The plots of (*F*(*R*)*hν*)^2^*vs. hν* for indirect allowed transition are shown in Fig. 5. It was found that the estimated value of *E*_g_ for the NiS_2_ layer was 1.19 eV, which is in agreement with reported values.^33,56^ The low *E*_g_ value would allow the utilization of this material in photocatalytic applications under solar radiation.^29,57^

### Photocatalytic activity measurements

**Fig. 6:**
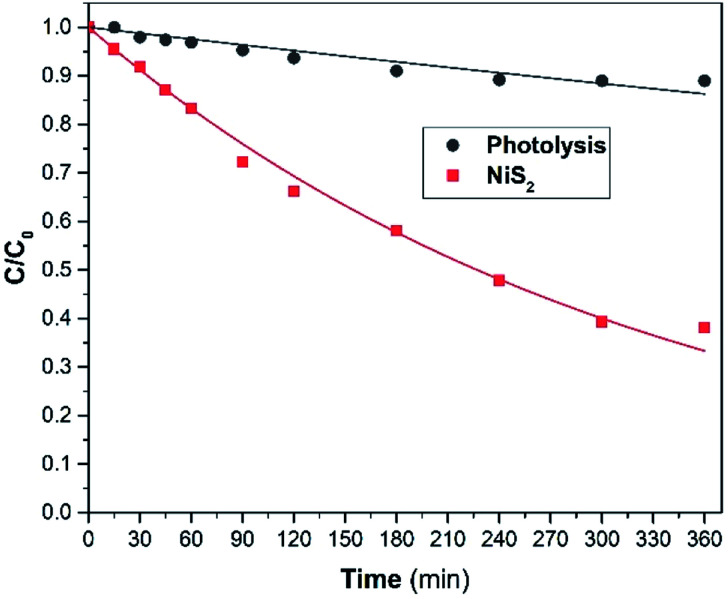
Photocatalytic performance of NiS_2_, phenol conc. = 10 mg L^−1^, and natural pH 6.

The photocatalytic activity performance of the NiS_2_ sample (5 cm^2^) was examined using phenol as the model organic pollutant, two NiS_2_ samples and at natural pH of 10 mg L^−1^ phenol. The variation of phenol relative concentration (*C*/*C*_0_) is offered in Fig. 6 with the matching values of the 1^st^ order apparent rate constants. Phenol presented insignificant photolysis under solar light. On the other hand, the rate of phenol photodegradation under solar light in the presence of the as-prepared NiS_2_ layer was improved. This is due to the presence of the as-prepared NiS_2_ slide, which absorbs solar light and photogenerates e^−^/h^+^ pairs utilized in photodegradation.

**Fig. 7:**
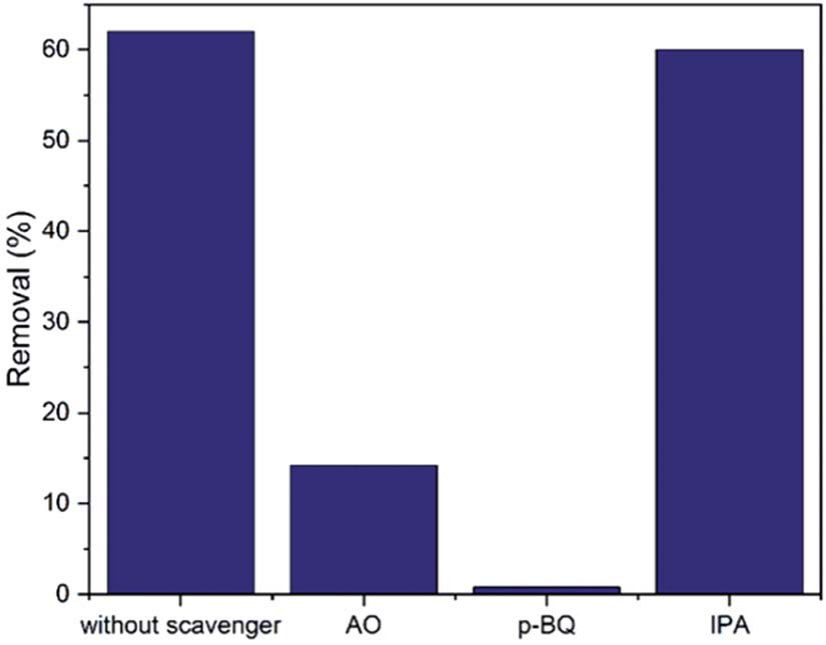
Effect of scavengers on phenol removal efficiency.

The main active species used in pollutant photodegradation are e^−^, h^+^, ˙OH and O_2_˙^−^. The active species produced by NiS_2_ are identified in Fig. 7. The active species identification was done by adding 1 mmol of each scavenger agent (AO, *p*-BQ and IPA) with 10 mg L^−1^ phenol and NiS_2_ layer compared to the experiment done without any scavenger. As shown in Fig. 7, the primary active species is O_2_˙^−^ and h^+^ is a secondary species, which are used as redox species in phenol photodegradation. Therefore, the proposed mechanism of the photocatalytic reactions is indicated by the following equations:

10 NiS_2_ + *hν* → NiS_2_(h^+^, e^−^)

11 NiS_2_(e^−^) + O_2_ → NiS_2_ + O_2_˙^−^

12 O_2_˙^−^ + h^+^ + phenol → photodegradation product

**Fig. 8:**
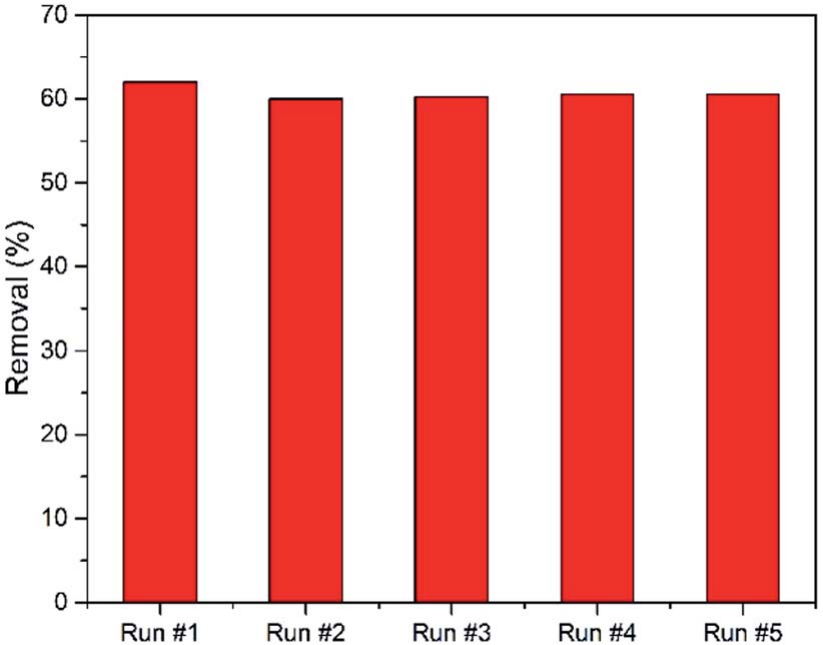
Reusability of NiS_2_, phenol conc. = 10 mg L^−1^, and natural pH 6.

On the other hand, the NiS_2_ reusability process is a very important issue, making the treatment process more economical. Fig. 8 shows a five cycle reusability test for NiS_2_ phenol photodegradation. The removal efficiency was slightly decreased after the first cycle. Thereafter, there was no change in the phenol removal efficiency after each cycle.

## Conclusion

A NiS_2_ layer with a nanoflake-like structure was successfully synthesized by a facile two-step growth process. The Ni(OH)_2_ layer was deposited on a glass substrate by chemical bath deposition, followed by a sulfurization process to obtain a single phase NiS_2_ layer. The XRD and Raman analysis confirmed the formation of single-phase NiS_2_. SEM revealed that the NiS_2_ layer consisted of overlapping nanoflakes. XPS measurements revealed that the observed peaks from Ni 2p and S 2p spectra were attributed to NiS_2_. The NiS_2_ displayed a narrow optical bandgap of 1.19 eV. The NiS_2_ nanoflake layer showed photocatalytic activity for the degradation of phenol under the irradiation of solar light at natural pH 6. The NiS_2_ nanoflake layer exhibited good solar light photocatalytic degradation of phenol with good stability and reusability. The as-prepared NiS_2_ layer can absorb solar irradiation and generate e^−^/h^+^ pairs. Hence, the NiS_2_ layer is a promising photocatalyst for the photodegradation of destructive organic pollutants.

## Author contributions

Mohammed M. Gomaa: conceptualization, data curation, formal analysis, investigation, methodology, writing – original draft. Mohamed H. Sayed: conceptualization, data curation, formal analysis, investigation, methodology, writing – original draft. Mahmoud S. Abdel-Wahed: conceptualization, methodology, data curation, formal analysis, investigation, writing – original draft. Mostafa Boshta: funding acquisition, project administration, resources, supervision, validation, writing – original draft.

## Conflicts of interest

The authors declare no competing interests

## Supplementary Material
